# Gold Plates with Rubber Attachments

**Published:** 1870-12

**Authors:** W. H. Eames


### SELECTED ARTICLES.

### ARTICLE VI.

### *Gold Plates with Rubber Attachments*

By W. H. EAMES, D. D. S.

Among the many different styles of artificial work at the present day, there is none, perhaps, which for beauty, strength and cleanliness, excels what is known to the profession as combination work: a gold base with teeth attached by means of rubber. This style of work, when neatly executed, and so constructed as to hide perfectly the means of attaching the teeth, will rank among the finest specimens of dental art. We will endeavor to describe three methods of constructing this style of work, suggested by different operators.

CASE.—A full upper denture. The plate is struck up and fitted in the usual manner. The teeth (in blocks or sections adapted for rubber work) are ground to the plate and articulated as for the ordinary rubber attachment. In the final arrangement or articulation of the teeth in the mouth, before finishing, they should be attached to the plate with a strong adhesive wax, or with gutta-percha, to avoid displacement in the subsequent steps of the process. Remove the plate from the mouth, oil the outside of the teeth and edge of the plate, in two sections, meeting at the median line in front. When these impressions or molds are sufficiently hard, they should be removed from the teeth and trimmed to about the one-half of an inch in thickness, so that the moisture can be easily driven off. As these moulds are friable when dry, it is well to place a strip or two of small wire one inch and a half in length, in the body of the mould, before the material sets, to prevent their breaking. When the moulds are thoroughly dry, form a shallow cup around them with putty or sand for the reception of the metal and cast in the zinc; to form a die, get up the counter die with lead in the usual manner. In the dies thus prepared, strike up the rim for the outside edge. The gold used for this purpose should be fine, and not less than No. 30 Stubb's guage.

If there is to be no opportunity to test the accuracy of the articulation by trial in the mouth, before the work is completed, a small plaster articulator should be made before the teeth are disturbed, showing their exact position on the plate. Having secured this, remove the teeth from the plate by warming it slightly, and solder on the rim. This is best done by placing one section in position, retaining it there by means of small wire clasps, placing one at each end and one in the centre; commence soldering at the heel or back end; having secured this point, pass on to the centre and secure a point there, and finally at the front. Now place the teeth, which are still attached to the wax, in position, and see if they fit nicely down the plate within the rim, if not, the position of the rim may be changed slightly to admit the teeth, and then the soldering completed; secure the opposite side in the same manner, uniting the ends in front. A very good means of securing a nice adaptation of the parts is obtained by placing the moulds in which the die for the rim is cast, in position on the plate, after removing the teeth and casting a little tinner's solder or even lead or tin, in the space formed by the gums of the teeth, thus forming a matrix which may be forced down inside the rim after the soldering is complete, bringing the rim out for the reception of the teeth. If this plan be adopted, one should be careful to cover the surface of the plate with carbon or some substance to prevent the metal adhering to it. Having completed the outside rim, place the teeth in position on the plate, and by means of the articulator or the patient's mouth, see that their position is correct. Secure them firmly to the plate with a stiff wax (composed of equal parts resin and beeswax); with a wax knife, trim out the wax to the lingual border of the teeth, leaving only enough to cover the heads of the pins, scarcely that; trim as near to a perpendicular with the surface of the plate as convenient, flowing the surface on evenly; oil and take a plaster impression of the lingual surface of the plate and teeth, or the wax inside the teeth; from the plaster model thus formed, get up a set of dies and strike up the rim for the inside. This rim should also be of fine gold, and be in two sections or a single piece; in thickness about No. 26 or 28, dependent on the case. The posterior ends of the outer and inner rims should be so formed as to be united around the end of the posterior block. When this inside rim is fitted nicely all along the border of wax, while the other border fits up nicely against the lingual surface or margin of the teeth. When the rim is thus fitted, place it in position and mark the position of the rivets, in the blocks, on the rim; remove the rim, and between the points indicated for the rivets, punch two holes with a plate punch, one above the other; prepare some small staples of gold wire; pass the ends through holes, leaving the loops to project about the length of the pins in the blocks; solder them in on the lingual surface, and dress off the ends. These loops form the means of attaching the rubber. Place the rim in position of the plate, and retain it there by melting some shellac around the edge; carefully remove the teeth; prepare several wire clamps, of such shape, that while the open end clasps tighly the free margin of the rim, the opposite end is brought up to a line with the edge of the plate. Having placed these clamps in position around the rim, prepare an investment of equal parts plaster and sand, and invest the palatine surface of the plate, embracing the closed end of these clamps. When the investment is hard, the shellac may be removed from the lingual surface of the rim and plate, the clamps retaining the rim in contact with the plate. Care should be taken that no plaster reach the plate, or rim, where the soldering is to be done. Heat up evenly and flow the solder. The plates should be clean, and the borax put only where it is necessary to flow the solder. When the soldering is completed, pickle the plate and place the teeth in position; and if the rim should not fit the teeth nicely, burnish it down against them at all points, leaving no space for the rubber to show itself; prepare some rubber in small strips; set the plate with the teeth on in the oven, or in some place where it will get quite hot; remove all the blocks; place a few strips of rubber underneath the posterior block, on one side; place the block in position, forcing the excess of rubber forward underneath the second block; place more rubber under this second block, and press it down in the same manner. Proceed in this way till all are in position; if any rubber shows on pressing the last block to its place, remove it (the block) and cut off the surplus rubber; when all are in position and no rubber in sight, imbed the the case in plaster in a vulcanizing flask, and vulcanize. Finish the same as any ordinary gold plate.

Another mode of accomplising the same end has been adopted by Dr. Hewitt, of this city. After preparing the plate for the reception of the teeth in the manner above described, he drills four or five holes through the plate, about one eight of an inch in diameter, along the ridge underneath the base of the teeth; to these holes he fits very accurately little plugs of gold of the same material as the plate; he then packs the rubber into these holes, forcing it into one till it appears at the next, and so on till the case is packed; he then drives these plugs into the holes in the plate; the rubber closing around them, and into the notches previously made in the plugs, holds them fast, after the case is vulcanized. In finishing the plate, the protruding ends of these plugs are dressed off, and if neatly done, they cannot be seen.

Another plan of which we have heard, is the same except that the rubber is packed through the holes, in the posterior end of the rim, and then these holes are closed in the same or similar way to that of Dr. Hewitt's. By having the teeth carved to suit special cases, a beautiful artistic effect can be produced both in and out of the mouth by this style of work.

When ordinary rubber block teeth are used, the case may have a stiff, artificial look; the shape and arrangement of the teeth may not be all that could be desired; still it is superior in many respects to any other style of gold work. —*Missouri Dental Journal.*

---

